# Influence of the Regime of Electropulsing-Assisted Machining on the Plastic Deformation of the Layer Being Cut

**DOI:** 10.3390/ma11060886

**Published:** 2018-05-25

**Authors:** Saqib Hameed, Hernán A. González Rojas, José I. Perat Benavides, Amelia Nápoles Alberro, Antonio J. Sánchez Egea

**Affiliations:** 1 Department of Mechanical Engineering (EPSEVG), Universitat Politécnica de Catalunya, Av. de Víctor Balaguer Vilanova i la Geltrú, 08800 Barcelona, Spain; hameeds@tcd.ie (S.H.); hernan.gonzalez@upc.edu (H.A.G.R.); amelia.napoles@upc.edu (A.N.A.); 2 Department of Electrical Engineering (EPSEVG), GAECE group, Universitat Politécnica de Catalunya, Av. de Víctor Balaguer Vilanova i la Geltrú, 08800 Barcelona, Spain; iperat@ee.upc.edu; 3 Department of Mechanical and Metallurgical Engineering, Pontificia Universidad Catolica de Chile, Av. Vicuña Mackenna 4860, Region Metropolitana 7820436, Chile; 4 Department of Mechanical Engineering, Aeronautics Advanced Manufacturing Center (CFAA), Faculty of Engineering of Bilbao, Alameda de Urquijo s/n, 48013 Bilbao, Spain

**Keywords:** electropulsing, machinability, chip compression ratio, current density, specific cutting energy

## Abstract

In this article, the influence of electropulsing on the machinability of steel S235 and aluminium 6060 has been studied during conventional and electropulsing-assisted turning processes. The machinability indices such as chip compression ratio ξ, shear plane angle ϕ and specific cutting energy (SCE) are investigated by using different cutting parameters such as cutting speed, cutting feed and depth of cut during electrically-assisted turning process. The results and analysis of this work indicated that the electrically-assisted turning process improves the machinability of steel S235, whereas the machinability of aluminium 6060 gets worse. Finally, due to electropluses (EPs), the chip compression ratio ξ increases with the increase in cutting speed during turning of aluminium 6060 and the SCE decreases during turning of steel S235.

## 1. Introduction

Studying the behaviour of metallic alloys during machining is very important in the manufacturing industry. The aspects of good machinability are usually low energy consumption, short chips, smooth surface finish and long tool life [1]. The high process efficiency can be achieved by minimizing the energy required to remove the material as plastically deformed chip [2]. The work done in the plastic deformation of the chip is influenced by the cutting speed through the chip temperature, dimensions of deformation zone adjacent to the cutting edge and velocity of deformation [3]. An increase in cutting speed tends to decrease of chip thickness and the region of plastic deformation becomes smaller which ultimately reduces the energy consumption [4]. It was generally observed that in metal cutting processes only 30–50% of energy is spent for useful work, while 25–60% of energy consumed during cutting is simply wasted [5]. Therefore, reducing the energy spent in metal cutting as much as possible by selecting properly the workpiece material, machining regime and process parameters is of great importance. Generally, the energy consumption in metal cutting processes is a function of cutting speed, feed rates and workpiece material [6]. The SCE increases as the hardness and mechanical strength of cutting materials increase [7]. The high cutting speed reduces the tool life of the most difficult-to-cut materials [8]. During turning of medium carbon steel, the SCE decreased by increasing the feed rate and depth of cut [9]. Fernandez-Abia et al. [10] analyzed that high cutting speeds reduce the cutting forces, which implies less power consumption and the chip thickness is significantly reduced, which implies low chip compression ratio ξ and high shear plane angle ϕ. It was generally observed that chip compression ratio ξ is the measure of plastic deformation of material which decreases with the increase in cutting speed [3].

During machining of ductile materials such as aluminium, a large tool chip contact and high chip compression ratio ξ increases the cutting forces, machining power and heat generation [1]. Materials with more ductility have higher degree of plastic deformation and high temperature of chip than that of less ductility during cutting [11]. Yousef et al. [12] suggested that an excessive increase in deformation rate may increase the machining forces in aluminium alloys. Becze et al. [13] indicated that the adiabatic temperature rise presented considerable heating of shear band which will decrease the flow stress of material. When the material passes through the primary deformation zone, the plastic deformation starts and is sheared at a rapidly increasing strain rates until reaches its maximum value [14]. Lee et al. [15] studied the procedure of determining the large strain distribution in the primary deformation zone by using particle image velocimetry (PIV) technique. They examined that the shear strain rate varies linearly with the cutting speed and the shear zone is narrower for higher shear strain rates. Wang et al. [16] used similar image processing technology to investigate the thermal softening behaviours in several metals alloys electrically assisted under tensile test. They found the current density threshold to achieve an effective thermal softening for each metallic alloy. Previously, they also published electrically-assisted micro-tension results to describe the thermomechanical behaviour of the AZ31 magnesium alloy under different current densities and, consequently, different joule effects [17]. As a result, the thermal softening was attributed only to the contribution of the joule effect and the change of the material resistivity. Following this topic, Sanchez et al. [18] introduced a thermomechanical model to describe the flow stress in an electropulsing wire drawing process and showed the microstructure changes associated to an ultra-fast annealing treatment that promotes a detwinning mechanism. All these experimental studies used low strain rates where it is easy to induce a high amount of energy to affect the material misconstruction; on the contrary, for high strain rates at high cutting speed in machining, the energy required to complete the process is low [19]. Accordingly, Gui et al. [20] investigated that, due to the increase in cutting speed, the temperature rise in primary shear zone decreases the shear strength of material which ultimately reduces the cutting forces. They also validated that decreasing the uncut chip thickness decreases the temperature in the primary shear zone which increases the shear strength of material and hence increases the SCE. Bakkal et al. [21] demonstrated that high cutting speeds significantly reduce the SCE of materials with low thermal conductivity and high thermal softening effect.

Electropulsing, as an instantaneous high energy input method, is recognized as a novel technique in metal cutting processes. Baranov et al. [22] discovered the effect of current pulses on metal cutting area which reduces the static force of cutting and hence the plastic deformation of metals. Recently, Brandt et al. [23] examined that, due to electric current, the reduction in cutting forces is higher for higher strength materials. Sanchez et al. [24] observed that the SCE and the power consumption are decreased when the turning process is assisted with EPs. In a previous study [25], it was found that, at lower feeds, when the higher current density is induced, the shear angle ϕ decreases and the chip compression ratio ξ increases during drilling process. There was no study or few attempts were made to evaluate the machinability of materials with respect to chip compression ratio ξ, shear plane angle ϕ and SCE while cutting with EPs during turning process.

The main purpose of the present study was to investigate the influence of EPs on the plastic deformation and energy consumption of materials such as Steel S235 and aluminium 6060 during turning process. Since the chip compression ratio ξ can be considered as the true measure of plastic deformation in metal cutting [3], and SCE can be used to compare the machinability of different materials, an experimental investigation was performed to measure the chip compression ratio ξ and SCE by using different cutting parameters to understand the behaviour of metallic materials when machined with and without pulses.

## 2. Experimental Setup

The turning process was carried out by using WEISS WMP280V-F round turning machine (ORPI SL, Zaragoza, Spain). A carbide tool DIN4980-ISO6 P20 fitted with diamond ground carbide inserts, rake angle of $6^{\circ }$ and nose radius of 0.2 mm was used and held by standard tool holder to machine the metallic bars. Commercial steel alloys (S235) and aluminium alloys (Al 6060) of 20 mm diameter were chosen as workpiece materials for test specimens. The chemical composition of the studied metallic materials is shown in Table 1 and mechanical properties of both alloys are listed in Table 2.

A polymeric material was used to electrically isolate the workpiece and tool holder from the lathe. The power consumed was continuously measured by a self made monophasic energy analyzer linked to the motor of the machine. A self made short duration electric pulse generator was developed to discharge multiple positive pulses. The machining parameters and the parameters of current pulses such as voltage, frequency and pulse duration, which were monitored by an osciloscope, are listed in Table 3.

**Figure 1:**
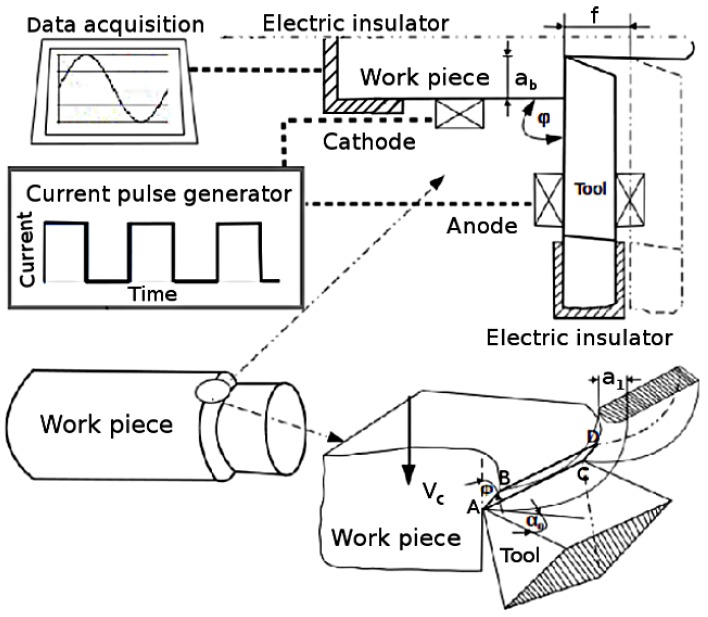
Schematic of electrically assisted turning process.

A schematic illustration of electrically assisted turning process is shown in Figure 1. The workpieces performed round turning process to make sure the surface was smooth and symmetric. The carbon clamps were attached with workpiece and connected on one side with cutting tool and on the other side with generator through wire. The turning operation was performed without EPs (conventional process). Subsequently, the same procedure was performed with EPs assisted process and it was confirmed that the workpiece was already in contact with the tool to avoid electro-erosion.

## 3. Results and Discussions

### 3.1. Current Density, Chip Thickness Ratio and Cutting Configurations

In machining, chip formation occurs by imparting shear within a narrow deformation zone called primary shear zone in which the effective strain rates are much larger [26]. The current density which is defined as current intensity through the cross sectional area of the material during cutting, can be considered as an important factor in changing the deformation resistance of material in primary shear zone. The shear cutting area is defined by the segment AB and the length of cutting edge BD ($a_{b}$) as shown in Figure 1. The segment AB is described as:

(1) $$AB=\frac{f}{sin(\phi )}$$

where *f* is the cutting feed (mm per rev) and ϕ is the shear plane angle (${}^{\circ }$).

The current density ($J_{e}$) in the primary deformation zone can be calculated by the following equation:

(2) $$J_{e}=\frac{I\cdot \sin(\phi )}{f\cdot a_{b}}$$

where $a_{b}$ is the depth of cut (mm) and I is the current intensity (A). The shear plane angle ϕ proposed in [27] can be expressed geometrically by chip compression ratio ξ as:

(3) $$\phi =\arctan\left(\frac{\cos\alpha_{0}}{\xi -\sin\alpha_{0}}\right)$$

Chip thickness coefficient is defined as a ratio ξ = $a_{1}/a$, where $a_{1}$ is deformed chip thickness and *a* is undeformed chip thickness, which in this case is the same value as the cutting feed. $\alpha_{0}$ is the rake angle which is $6^{\circ }$ for the particular tool used during experiments. Chips were collected randomly at the end of each cutting test to measure the thickness of the chip. The chip thickness was measured by using micrometer with 0.01 mm precision. At least five measurements were taken to get the average chip thickness. The spindle velocity was measured in rpm with a conventional tachometer. Table 4 gives the values of current densities, chip compression ratio ξ and shear plane angle ϕ with and without pulses as a function of cutting feed, cutting speed and depth of cut for steel S235.

It can be seen in Table 4 that chip compression ratio ξ decreases with the increase in cutting feed and cutting speed during turning of steel S235. In addition, the shear plane angle ϕ increases as chip compression ratio ξ decreases. However, as compared to conventional turning process, the values of chip compression ratio ξ is lower during electrically assisted turning process. The low values of chip thickness ratio ξ (high ϕ values) mean low shear strain in the shear plane [28]. Since chip compression ratio ξ is a measure of the plastic deformation of materia, which decreases with the increase in cutting speed [3], an increase in cutting speed leads to a decrease of plastic deformation in chip formation zone. It was generally observed that the chip thickness decreased as the cutting speed increased and the region of plastic deformation becomes smaller which ultimately reduces the energy consumption [4].

The current density decreases with the increase in cutting feed during electrically assisted turning process of steel S235 as shown in Table 4. However, the results are in agreement with the previous study [25], in which current density decreases with the increase in cutting feed during electrically assisted drilling process. As the chip formation zone decreases with increase cutting speed, this may be the reason why current density values are higher at high cutting speeds for steel S235 during electrically assisted turning process. Table 5 gives the values of chip compression ratio ξ, shear plane angle ϕ and current densities with and without pulses as a function of cutting feed, cutting speed and depth of cut for aluminium 6060. Table 5 shows that chip compression ratio ξ decreases with increase in cutting feed and increases with the increase in cutting speed. In addition, shear plane angle ϕ increases with the increase in cutting feed and decreases with the increase in cutting speed. The high values of chip compression ratio ξ mean large amount of strain in shear plane [28]. An increase in the cutting speed leads to an increase in the temperature of the chip so its plastic deformation increases [3]. Hence, during turning of aluminium 6060, an increase in cutting speed tends to increase chip compression ratio ξ which indicates severe plastic deformation in the chip formation zone. It is also seen in Table 5 that, during electrically assisted turning process, the values of chip compression ratio ξ are higher and that of shear plane angle ϕ are lower than conventional turning process in aluminium 6060. In addition, the current density decreases with the increase in cutting speed while increases with the increase in cutting feed.

**Figure 2:**
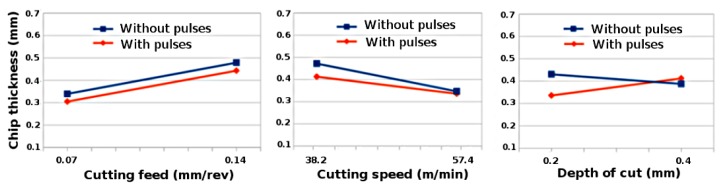
Variation of chip thickness with cutting feed, cutting speed and depth of cut while turning steel S235 with and without pulses.

**Figure 3:**
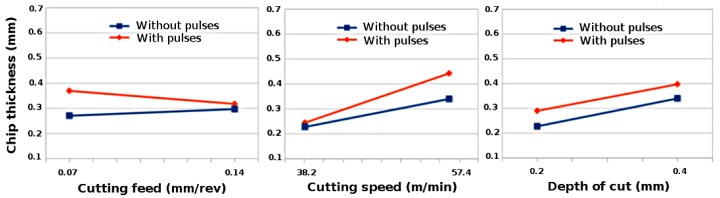
Variation of chip thickness with cutting feed, cutting speed and depth of cut while turning aluminium 6060 with and without pulses.

Figure 2 demonstrates the variation of chip thickness with cutting feed, cutting speed and depth of cut for steel S235 by using factorial analysis. The chip thickness increases with the increase in cutting feed and decreases with the increase in cutting speed. The increase in undeformed chip thickness with increasing feed rates will result in increase of shear plane area [29]. The reduction in chip thickness will result in shorter shear plane and longer shear plane is associated with thicker chip produced during the cutting process [30]. However, depth of cut has very little effect on chip thickness during conventional turning process but chip thickness increases with the increase in depth of cut during EPs assisted turning process. Figure 3 shows that the cutting feeds have very little effects on chip thickness, however chip thickness increases with the increase in cutting speed and depth of cut during turning of aluminium 6060. As compared to conventional process, the chip thickness values are higher during electrically assisted turning process. When the chip thickness increases, the chip compression ratio ξ also increases which means plastic deformation of material increases.

### 3.2. Specific Cutting Energy (SCE)

The machinability of materials can be estimated by comparing the SCE during EPs assisted and conventional turning processes. The SCE is the quotient between the cutting power consumption and the material removal rate as described in the previous work [25]. The material removal rate, $Q_{c}$, is defined by the following equation:

(4) $$Q_{c}=V_{c}\cdot f\cdot a_{b}\left(1-\frac{a_{b}}{D}\right)$$

where $V_{c}$ is cutting speed (m/min) and D is the workpiece diameter (mm).

The mechanical power consumed was evaluated indirectly during turning by measuring the active electrical power consumed by motor of lathe Pa. The device that measured the active electrical power was constructed with an analog chip, multiplier of 4 quadrants AD633, which gives the product of supply voltage and current consumed. This signal was digitalized with digital analog converter. The mechanical power delivered by the motor multiplied by mechanical efficiency of turning machine allows obtaining cutting power. The mechanical efficiency was not known. The relationship between the active electrical power and mechanical power was also unknown. To obtain the relationship between the cutting power and active electrical power, an experiment was carried out in which a mechanical load was imposed by dynamometer brake and active electrical power was measured for different spindle speeds. With the data of experiment, a linear regression was obtained giving an expression that allowed to relate the active electrical power with mechanical power consumed by the spindle of lathe. Finally, the cutting power obtained as the difference between the mechanical power during cutting and mechanical power without load in conventional turning process was then compared with the cutting power obtained in electrically assisted turning process.

**Figure 4:**
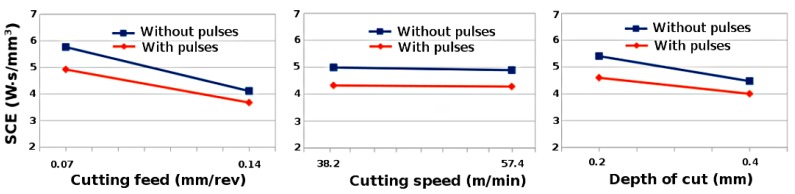
Variation of the SCE respect to the cutting feed, cutting speed and depth of cut while turning steel S235 with and without electropulsing.

Figure 4 show that the SCE decreased with the increase in cutting feeds and depth of cut for steel S235 by using factorial analysis. It was observed that the effect of cutting speed on SCE is negligible for steel S235. However, the SCE decreased due to the application of EPs during cutting. The reduction in SCE was observed: 7% for high feeds and 14.6% for low feeds; 12.4% for high cutting speed and 10.5% for low cutting speed; and 10.5% for high depth of cut and 12.1% for low depth of cut. Hence, due to thermal contribution of EPs, the deformation resistance decreases [31], which ultimately decreases the SCE and improves the plasticity of material during EP assisted turning process. The results are also in agreement with Sánchez et al. [24] and Hameed [25] where the SCE decreases due to the application of EPs during machining processes.

**Figure 5:**
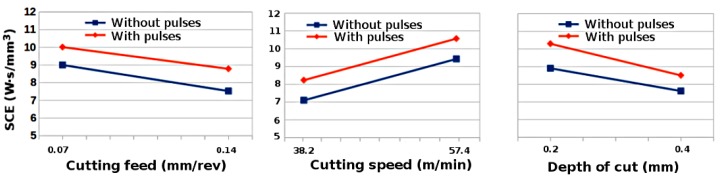
Variation of SCE with cutting feed, cutting speed and depth of cut while turning aluminium 6060 with and without pulses.

Figure 5 presents that SCE decreased with the increase in cutting feed and depth of cut for aluminium 6060 by using factorial analysis. However, as compared to steel S235, a different trend was observed when the cutting speed increased the SCE also increased. It is also noted that SCE increased due to the application of EPs during cutting. Since aluminium 6060 has higher thermal conductivity and less thermal softening effect as compared to steel S235, at higher cutting speeds and due to the application of EPs, the strain rate in the shear zone is expected to be high. Thus, more heat energy will be generated and time for heat dissipation decreases, which ultimately increases the temperature [32]. In addition, an excess of electrons can increase the flow stress during superplastic deformation of aluminium alloy [33]. Hence, due to increase of temperature and flow stress, the plastic deformation of material increases which increases the SCE in aluminium 6060. Furthermore, it can be observed that, by increasing the cutting speed for steel S235, the chip thickness decreases and current density increases, while for aluminium 6060, by increasing the cutting speed, chip thickness increases and current density decreases, due to which SCE increases during turning of aluminium 6060 due to the application of EPs. It is assumed that area of deformation zone decreases by increasing the cutting speed for steel S235 and increases by increasing the cutting speed for aluminium 6060. Thus, it is expected that, as compared to steel S235, plastic deformation increases with the increase in cutting speed for aluminium 6060 because of increase of temperature in the chip formation zone.

## 4. Conclusions

The effect of electropulsing was observed during turning of steel S235 and aluminium 6060. Correlation among chip compression ratio ξ, shear plane angle ϕ, current density, chip thickness and specific cutting energy was obtained by using different cutting parameters for both conventional and electrically assisted processes. The main findings can be summarized as following:

- By increasing the cutting speed, the chip compression ratio ξ decreases and shear plane angle ϕ increases during conventional turning of steel S235. However, increase of cutting speed seems to have no effect on chip compression ratio ξ and shear plane angle ϕ during electrically assisted turning of steel S235. In contrast, chip compression ratio ξ increases and shear plane angle ϕ decreases with the increase in cutting speed while turning of aluminium 6060 during conventional and electrically assisted processes.
- The current density increases with the increase in cutting speed and decreases with the increase in cutting feed for steel S235. However, the current density has high values at higher feeds and decreases with the increase in cutting speed for aluminium 6060.
- The SCE decreases with the increase in cutting speed and depth of cut for both conventional and electrically assisted turning of steel S235. In addition, as compared to conventional process, the SCE values are lower during electrically assisted turning of steel S235. However, during conventional turning of aluminium 6060, the SCE decreases with the increase in cutting feed and depth of cut, while increases with the increase of cutting speed. In addition, the SCE values are higher during electrically assisted turning of aluminium 6060 as compared to conventional process.
- The electrically assisted turning processes tends to have influence in improving the machinability of steel S235 but for aluminium 6060, the machinability seems to be poor, which is probably due to its high ductility and higher degree of plastic deformation as compared to steel S235. Further investigations are required to further analyze the effect of EPs on machinability of materials by studying the cutting parameters and chip morphology.

## Figures and Tables

**Table 1:** Chemical composition of studied materials.

Aluminium 6060	% Al	% Zn	% Mg	% Cu	% Fe	% Si	% Mn	% Cr
	balance	0.15	0.35	0.1	0.1	0.3	0.1	0.05
Steel S235	% C	% P	% Cu	% Fe	% Si	% Mn	% S
	0.24	0.04	0.20	balance	0.04	0.9	0.05	

**Table 2:** Mechanical properties of studied materials.

Materials	ρ	$C_{p}$	*E*	α	*D*	Hardness
	(Ωm)	(J/Kg ${}^{\circ }$C)	(GPa)	(${}^{\circ }$C${}^{-1}$)	(Kg/m^3^)	(95% Data Interval)
Aluminium 6060	$3.2\times 10^{-8}$	900	70	$23\times 10^{-6}$	2700	71.4±1.8 (HB)
Steel S235	$1.42\times 10^{-7}$	470	190	$12\times 10^{-6}$	7800	62.8±1.5 (HRB)

**Table 3:** Turning and electropulsing operation parameters.

Material	Spindle	Cutting	Depth of	Current	Pulse	Frequency
	Speed	Feed	Cut	Intensity	Duration	
	(rpm)	(mm/rev)	(mm)	(A)	(μs)	(Hz)
Aluminium 6060	600/900	0.07/0.14	0.2/0.4	140	250	300
Steel S235						

**Table 4:** Values of chip compression ratio, shear plane angle and current densities as a function of cutting feed and cutting speed for steel S235.

Material	Workpiece	Cutting	Cutting	Depth	Compression	Shear Plane	Current
	Diameter	Feed	Speed	of Cut	Ratio	Angle Density
**Steel**		**(f)**	**($V_{c}$)**	**($a_{b}$)**	**(ξ)**	**(ϕ)**	**($J_{e}$)**
**S235**	**(mm)**	**(mm/rev)**	**(m/min)**	**(mm)**		**(** ${}^{\circ }$ **)**	**(A/mm^2^)**
Without pulses		0.07	38.213	0.2	6.357	9.049	-
		0.14	38.213	0.2	4.321	13.356	-
		0.07	57.461	0.2	3.685	15.663	-
	20	0.14	57.461	0.2	2.957	19.484	-
		0.07	38.213	0.4	5.257	10.964	-
		0.14	38.213	0.4	3.343	17.262	-
		0.07	57.461	0.4	4.085	14.123	-
		0.14	57.461	0.4	3.057	18.856	-
With pulses		0.07	38.213	0.2	3.100	18.599	3190
		0.14	38.213	0.2	2.492	23.007	1954
		0.07	57.461	0.2	3.171	18.186	3121
	20	0.14	57.461	0.2	2.557	22.449	1909
		0.07	38.213	0.4	4.214	13.697	1184
		0.14	38.213	0.4	4.228	13.651	590
		0.07	57.461	0.4	4.128	13.982	1208
		0.14	57.461	0.4	3.378	17.081	734

**Table 5:** Values of chip compression ratio, shear plane angle and current densities as a function of cutting feed and cutting speed for Aluminium 6060.

Material	Workpiece	Cutting	Cutting	Depth	Compression	Shear plane	Current
	Diameter	Feed	Speed	of Cut	Ratio	Angle	Density
**Aluminium**		**(f)**	**($V_{c}$)**	**($a_{b}$)**	**(ξ)**	**(ϕ)**	**($J_{e}$)**
**6060**	**(mm)**	**(mm/rev)**	**(m/min)**	**(mm)**		**(${}^{\circ }$)**	(**A/mm${}^{2}$)**
Without pulses		0.07	38.213	0.2	2.371	24.571	-
		0.14	38.213	0.2	1.214	45.312	-
		0.07	57.461	0.2	3.771	15.375	-
	20	0.14	57.461	0.2	2.214	26.287	-
		0.07	38.213	0.4	4.028	14.372	-
		0.14	38.213	0.4	2.085	27.866	-
		0.07	57.461	0.4	5.285	10.877	-
		0.14	57.461	0.4	2.971	19.600	-
With pulses		0.07	38.213	0.2	3.514	16.526	2844
		0.14	38.213	0.2	1.114	48.470	3743
		0.07	57.461	0.2	5.001	11.515	1996
	20	0.14	57.461	0.2	2.914	19.999	1709
		0.07	38.213	0.4	3.971	14.583	1259
		0.14	38.213	0.4	2.085	27.321	1147
		0.07	57.461	0.4	8.628	6.583	573
		0.14	57.461	0.4	2.914	19.999	854
